# MC4R biased signalling and the conformational basis of biological function selections

**DOI:** 10.1111/jcmm.17441

**Published:** 2022-07-11

**Authors:** Zekun Liu, Victor J. Hruby

**Affiliations:** ^1^ Department of Chemistry and Biochemistry The University of Arizona Tucson Arizona USA

**Keywords:** Biased signalling, Conformational studies, MC4R, MC4R drug design, MC4R structure

## Abstract

The MC4R, a GPCR, has long been a major target for obesity treatment. As the most well‐studied melanocortin receptor subtype, the evolutionary knowledge pushes the drug development and structure–activity relationship (SAR) moving forward. The past decades have witnessed the evolution of scientists' view on GPCRs gradually from the control of a single canonical signalling pathway via a bilateral ‘active‐inactive’ model to a multi‐state alternative model where the ligands' binding affects the selection of the downstream signalling. This evolution brings the concept of biased signalling and the beginning of the next generation of peptide drug development, with the aim of turning from receptor subtype specificity to signalling pathway selectivity. The determination of the value structures of the MC4R revealed insights into the working mechanism of MC4R activation upon binding of agonists. However, new challenge has risen as we seek to unravel the mystery of MC4R signalling selection. Thus, more biased agonists and ligands with representative biological functions are needed to solve the rest of the puzzle.

## 1 INTRODUCTION

The melanocortin 4 receptor (MC4R) is a rhodopsin‐like GPCR with multiple physiological functions. It appears all over the human body, but centrally in the hypothalamus, playing important roles in energy homeostasis and appetite control.^1^ Previous research has found the association of MC4R mutations with obesity: a wide variety of heterozygous loss‐of‐function mutations cause a morbid early‐onset obesity syndrome or hyperphagia whereas gain‐of‐function mutations that increase receptor activity are associated with leanness.^2^ In this respect, MC4R has been a prime pharmaceutical target for obesity. Studies have shown that food intake leads to the secretion of α‐melanocyte‐stimulating hormone(α‐MSH) which activate the MC4R and downstream signalling to generate neuronal impulse of satiety feeling^3^ and MC4R is also highly related to energy homeostasis and expenditure.^4^ In addition, MC4R activation is also found to be linked to blood pressure, heart rate and libido generation, making it a popular and important drug target.^5^

In recent decades, scientists have engaged in developing MC4R selective agonists, and many were created.^6^ However, very few of them gave satisfying in vivo drug effects for different reasons. Early problems come from the selectivity of ligands binding to MC4R. Because melanocortin receptors (MCRs) have 5 subtypes and they structurally resemble each other a lot.^7^ Among the 5 subtypes, MC2R is always excluded from the MC4R study because it cannot be activated by endogenous melanocyte‐stimulating hormones(MSHs) as other subtypes are, and it functions more as adrenergic receptors.^8^ In addition to the structural similarity between MCR subtypes, all MCRs belong to the GPCR family which are highly dynamic membrane‐integrated proteins. This high level mobility makes them own non‐fixed conformations and can be induced to fit different ligands upon binding,^9^ which adds difficulty in drug design based on conformations because it has been very hard to obtain a MCR crystal structure. Thus, for a long time, MCR drug design had been empirical. Development of melanotropins started from mimicking the natural agonists MSHs, and later on, many more derivatives were created via versatile modifications exerted on these early mimics.^10^ Even though some of them already showed acceptable MC4R selectivity and preclinical efficacy, their clinical trial results for obesity treatment were not satisfying because of severe side effects including increased blood pressure, increased heart rate, vomiting, nausea and sex arousal.^11^, ^12^, ^13^, ^14^, ^15^ However, these observations pushed forward the MC4R signalling research, and it was found recently that the signalling activated by MC4R ligands can actually be biased.^16^ In this review, we have summarized the most recent findings about MC4R signalling capabilities and the structural basis related to these properties.

## 2 BIASED SIGNALLING IS WIDELY EXISTENT OVER GPCRs

As a GPCR, MC4R shares a lot of similar characteristics as other GPCRs. GPCRs have seven transmembrane domains (TM) which are highly conserved transmembrane alpha helices. The name GPCR is short for G protein‐coupled receptor because they convey signals across biological membranes via interaction with intracellular guanine nucleotide‐binding proteins (G proteins). This superfamily comprises approximately 2% of all proteins encoded in the human genome and is the target of a substantial portion of current pharmaceuticals.^17^ G proteins consist of heterotrimeric(Gαβγ) subunits which behave as transducers to switch between inactive/active states upon GDP/GTP exchange to initiate multiple intracellular signalling pathways. While GTP exchange factors (GEF) promote activation, GTP hydrolysis turns them off by the GTPase domain which exists in monomeric G proteins and Gα subunit of the heterotrimers. The G protein has approximately 20 α, 6 β and 12 γ subunits.^18^ Activating GPCRs will recruit specific G protein subtypes and trigger specific downstream signalling controlled by this G protein. Even though for a long time it had been commonly regarded that one specific receptor always applies the preferred/dominated signalling, it is now well accepted that a GPCR signalling can be biased in 2 different dimensions. The first dimension is that a single agonist can be multipotent and activate multiple signalling cascades through binding to different receptor subtypes. For example, epinephrine can activate Gα_s_, Gα_i_ and Gα_q_ through binding to β, α2 and α1 adrenoceptors, which represents the different response that different receptors to the same ligand.^19^

The second dimension is what we want to discuss here. This dimension describes how different ligands can induce different signalling cascades upon binding to a single receptor, which is significant to the selection of MC4R‐controlled multiple biological functions. For example, MC4R can selectively adopt one or several Gα_s_, Gα_q_, Gα_i_ and other G protein‐directed pathways when binding with specific ligands, depending on how the active conformation is induced by a ligand and how this induced conformation is suitable for recruiting a specific G protein subtype. The signalling biased by different ligands is not necessarily to be ‘one or another’. Some agonists show greater efficacy and potency to activate one pathway among all the downstream repertoire of the same receptor, indicating the bias. For instance, the relative order of potency for the pituitary adenylyl cyclase‐activating polypeptides (PACAP‐27 and PACAP‐38) in terms of activation of adenylyl cyclase (AC) and phospholipase C (PLC) is reversed. The former is more potent for activation of AC, while the latter displays greater potency for the PLC pathway.^20^ Likewise, structurally diverse α2 adrenoceptor (α2AR) agonists, catecholamines and phenol amines, display different orders of potency for Gα_i_, Gα_s_ or Gα_q_ activation.^21^ Such ligand‐specific divergence in receptor‐mediated activation of downstream pathways is also reported for class B and class C members of the GPCR superfamily.^22^

## 3 THE NATURALLY OCCURRING BIASED SIGNALLING CONTROLS MC4R ACTIVITY IN VIVO

**FIGURE 1:**
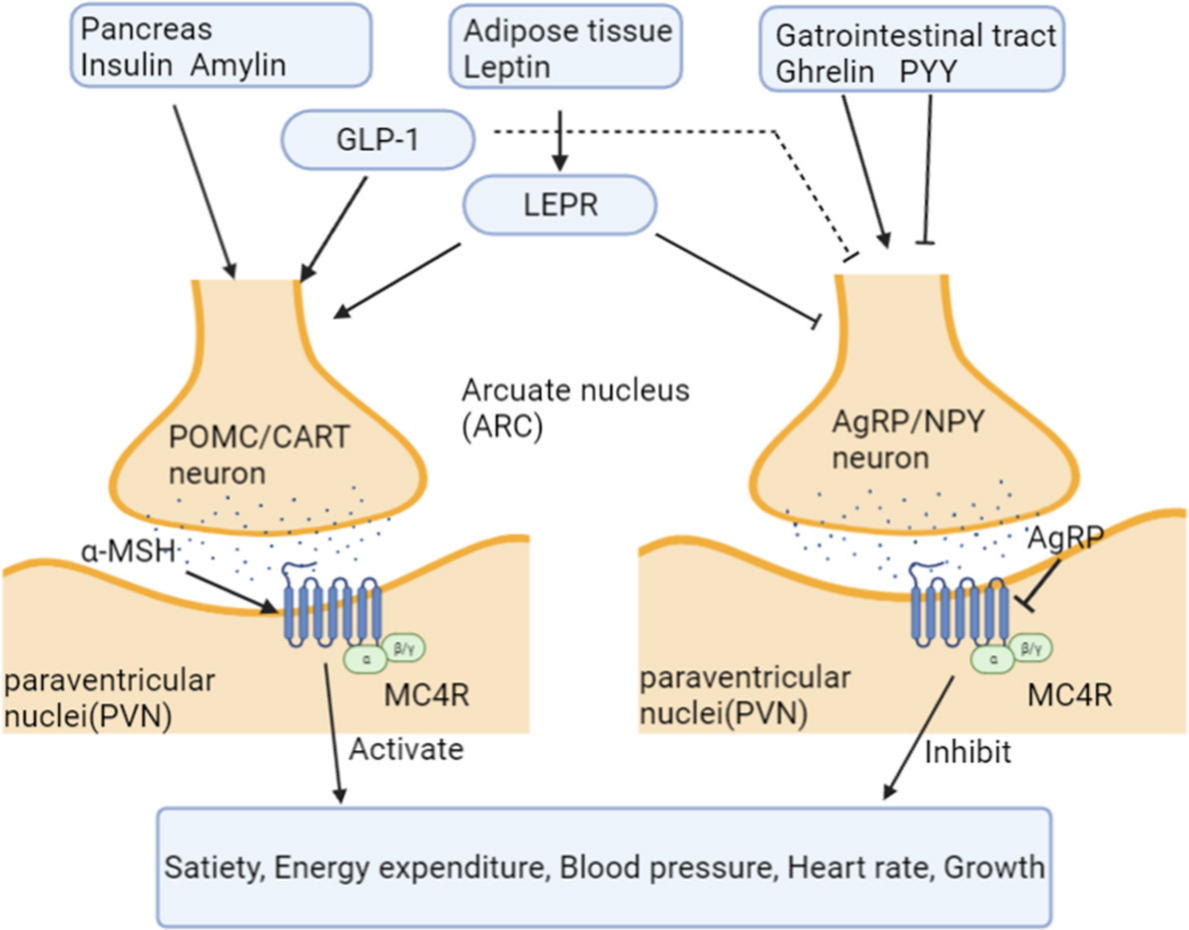
Melanocortin Signalling Cascade. Various peripheral signals, such as insulin, amylin, leptin, ghrelin, peptide YY (PYY) and glucagon‐like peptide 1 (GLP‐1), regulate the functions of secretion neurons in the hypothalamic arcuate nucleus (ARC). After taking food, pro‐opiomelanocortin (POMC) expressing neurons are activated leading to the secretion of melanocyte‐stimulating hormone (MSH). MSH can activate the melanocortin‐4 receptor (MC4R) expressed in the paraventricular nucleus (PVN), regulating satiety, energy expenditure, blood pressure and growth. In parallel, agouti‐related peptide (AgRP)‐expressing neurons, which are localized in the ARC, can be regulated by the hunger signals as well as inhibited by some satiety signals after food intake for example leptin and GLP‐1. AgRP can inhibit MC4R signalling and thus inhibits the generation of satiety. The indirect regulation is shown in dashed line

The first mutations in the MC4R were found about 15 years ago in the hypothalamus of the obese patients,^23^ giving evidence that MC4R could have the function of body‐weight regulation. More recently, it was clearly demonstrated that the MC4R is activated by the POMC‐derived neuropeptides α‐MSH and β‐MSH and blocked by Agouti‐related peptide (AgRP).^24^ The function of these neurons is modulated by signals from adipose tissue or the gut, such as leptin, ghrelin and NPY and finally goes up to the hypothalamus taking effect at neurons including those with MC4R expression. It is worth noticing the use of GLP‐1 receptor agonists in control of appetite during the past decades.^25^, ^26^ Throughout the hypothalamus, the GLP‐1 receptor is present particularly in the paraventricular nucleus (PVN), dorsomedial hypothalamus (DMH) and the arcuate nucleus (ARC), with a greater density on pro‐opiomelanocortin (POMC) neurons (anorexigenic neurons) than on the agouti‐related peptide (AgRP)/neuropeptide Y (NPY) neurons (orexigenic neurons).^27^ It is fairly clear that GLP‐1R activation directly stimulates POMC/CART neurons and indirectly inhibits neurotransmission in AgRP/NPY neuron via GABA‐dependent signalling.^28^ Interestingly, the GLP‐1R agonist liraglutide and MC4R agonist setmelanotide had additive effects on glycaemic control, weight loss and cholesterol metabolism, indicating their independent metabolic effects.^29^ Appetite is regulated by a complicated web of hormonal signals, while MC4R locates at the end of this regulation route. Therefore, MC4R has been considered as a potential target for obesity treatment. Figure 1 shows the MC4R regulation of appetite in the neuron system.

MC4R was first cloned in 1993 and shown to be coupled to the stimulatory G protein Gα_s_. For a long time, the MC4R signalling was known to be single, and the working mechanism of which was known to be simple as ‘lock and key’, that the binding of ligand works as a key inserted into the receptor lock, turning it from ‘off’ state to ‘on’ state. This tuning will then release G protein (Gα_s_) and lead to the cAMP‐PKA cascade. So, the appetite control by MC4R used to be regarded as a result of Gα_s_ signalling and a lot of drugs were developed with the Gα_s_‐cAMP‐PKA based biological assays. However, even though most of the human made MC4R agonists exhibit positive appetite inhibition upon activating MC4R, they also possess severe cardiovascular side effects as well.^30^ In addition, more and more evidence was found that some of the hyperphagia patients have consecutively active MC4R mutants and increased cAMP, which challenged the conventional idea of the control of appetite by Gα_s_ signalling.^31^ Later, more and more studies together confirmed that energy expenditure is the mainly mediated biological function through Gα_s_ signalling.^32^ For example, Chen et al.^33^, ^34^ found cases of central nervous (CNS) system‐specific Gα_s_ deficiency that triggers a specific defect in energy expenditure without an effect on food intake, indicating that appetite control and energy homeostasis are controlled by different signalling pathways.

MC4R‐biased signalling naturally occurs. It is the less common one in the GPCR family that MC4R is controlled by endogenous agonist MSHs and inverse agonist AgRP.^35^ GPCRs perform basal level activity under static state and has increased activity when binding with agonist^36^, while inverse agonist can even lower the basal level activity.^37^ As an MC4R inverse agonist, AgRP binding not only antagonizes the MC4R, but also decreases the basal level MC4R activity which is independent of melanotropins.^38^ This indicates MC4R's potential to initiate signals more than Gα_s_‐PKA cascade. The study by Buch etc.^39^ demonstrated that AgRP works as a biased agonist to selectively activate Gα_i_ protein in GT1–7 cells expressing MC4R. The incorporation of GTPγS35 to G protein/MC4R complex was measured and they found AgRP could promote the incorporation of GTPγS35 which can at the same time be blocked by pertussis toxin (PTX), suggesting that AgRP activates Gα_i_ protein in addition to simultaneously blocking Gα_s_ signalling. Apparently, the conventional lock‐key model that represents single GPCR function does not apply to explain the naturally occurring biased signalling phenomena. Thus, a new MC4R activation mechanism has been more and more proposed and accepted in recent years: G protein is recruited after GPCR activation instead of pre‐coupled and the recruitment is highly dependent on the induced conformation due to ligand binding. That is to say, GPCR activation occurs through allosteric coupling^40^, the propagation of conformational changes from the extracellular ligand‐binding pocket to the intracellular G protein‐binding interface and ligand‐binding site changes can allosterically regulate GPCR signalling and engender functional selectivity.^41^ In such alternative G protein coupling model, α‐MSH‐induced anorexigenic stimulus through MC4R is initiated by one of the MC4R active conformations which prefers Gα_s_ binding, whereas AgRP‐induced orexigenic stimulus is initiated by another MC4R active conformation which triggers Gα_i_ signalling.^42^ Meanwhile, AgRP was shown to inhibit excitation of hypothalamic neurons in a PTX‐sensitive manner, which further demonstrated that it is Gα_i_ signalling instead of other pathways non‐related to MC4R.^43^

## 4 FOOD INTAKE IS SPECIALLY CONTROLLED BY MC4R‐BIASED SIGNALLING, BRINGING IMPACT TO CONVENTIONAL MC4R DRUG DESIGN FOR HYPERPHAGIA AND OBESITY

Food intake, as another aspect of maintaining human body energy balance, is shown to be controlled by an integrated system. Adipose tissue, gastrointestinal organs, pancreas and so forth can all participate in the regulation of this behaviour via the secretion of different hormones including leptin, ghrelin, amylin, somatostatin and so on.^44^, ^45^ In earlier times, food intake was regarded as being controlled by a MC4R canonical pathway, which is Gα_s_‐PKA pathway; however, more and more studies found it could be independent from this pathway. Li et al.^46^ found that the PLC activator Gα_q_ and Gα_11_ knockout in the paraventricular nucleus (PVN) leads to severe hyperphagic obesity, increased linear growth and inactivation of the hypothalamic–pituitary–adrenal axis. However, this knockout does not affect the energy expenditure or glucose metabolism. Moreover, even in the animals lacking Gα_s_, the Gαq/11 knockout can still inflict the loss of appetite inhibition, indicating that the appetite inhibition is regulated by Gαq/11 instead of Gα_s_. Interestingly, when lacking Gα_s_, blood pressure response to the MC4R activation was lost in animals, building the relation of this effect to the MC4R mediated Gα_s_ signalling.^46^ These findings together gave evidence of the cardiovascular side effects brought by drugs designed based upon Gα_s_ signalling via the MC4R.

**FIGURE 2:**
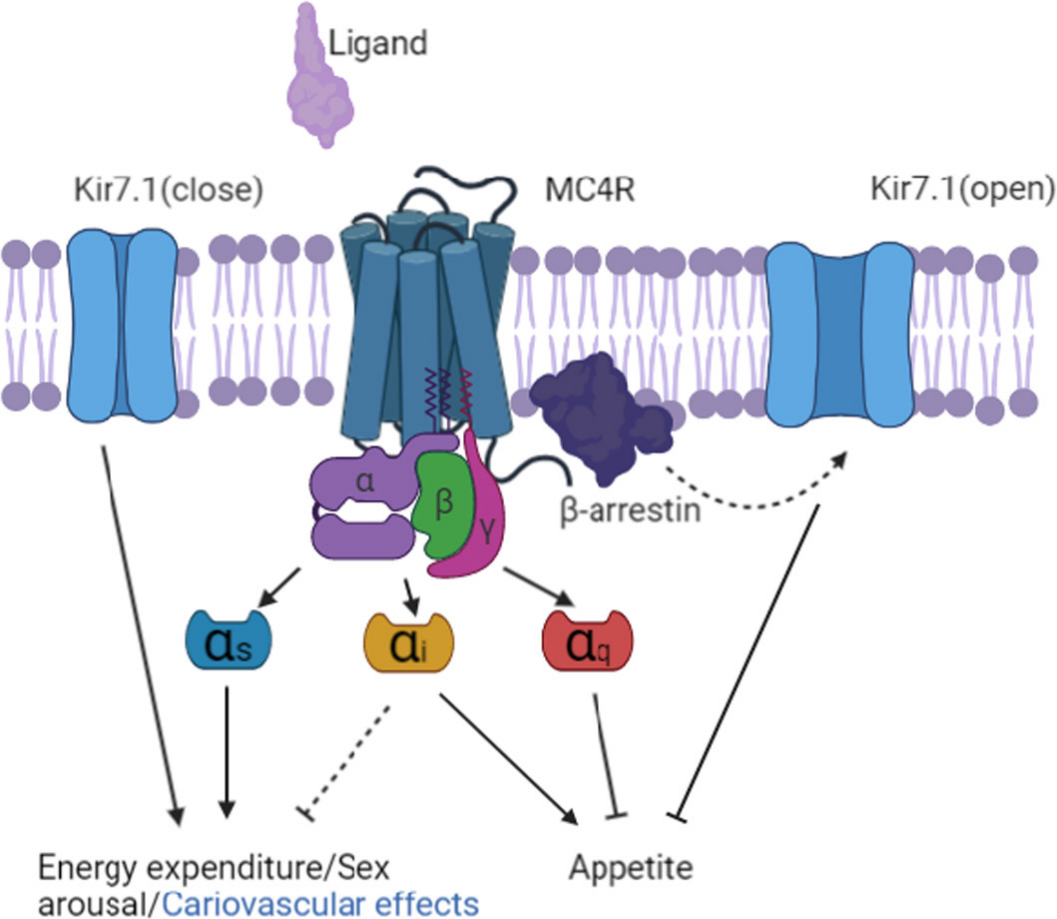
Therapeutic relevance of biased signalling at MC4R. The MC4R is able to recruit Gα_s_, Gα_q_, Gα_i_ and β‐arrestin and to couple to Kir7.1. Specially, activation of Gα_s_ and closure of Kir7.1 lead to a negative energy balance with side effects. Ligands specially activate Gα_q_ and potentially β‐arrestin and closure of Kir7.1, resulting in a negative energy balance without cardiovascular side effects

Apart from the MC4R, the inward rectifier potassium channel Kir7.1 is also related to appetite control due to its ability to depolarize/hyperpolarize the paraventricular nucleus of the hypothalamus (PVH) so that further signals for example satiety/food intake can be projected downward to other neurons.^4^, ^47^ In CNS regions outside of the PVH, depolarization is dependent on potassium channels which are regulated by Gβγ subunits from G protein.^48^ However, previous data from hypothalamic slice preparation indicated that depolarization of MC4R activated PVH is regulated by Kir7.1 with a G protein independent manner. In their research, Langroudi et al.^47^ applied the GDPβS (inhibitory GDP analogue) and other G protein signalling inhibitors such as gallein (Gβγ blocker) into the system, and these were ineffective in blocking the depolarization. This phenomenon indicates that the potassium channel activity in PVH neurons is not related to G protein functions. In their comparison of the roles of α‐MSH and AgRP, they found that α‐MSH not only increased intracellular cAMP levels through _Gαs_ signalling, but also decreased K^+^ efflux through potassium channel Kir7.1 to achieve the depolarization. However, AgRP increases K^+^ efflux but does not trigger Gα_s_ signalling at the same time. This evidence further showed AgRP's signalling bias on MC4R over Gα_s_, which is related to Kir7.1 regulation to generate a hyperpolarization. When building the connection between MC4R and Kir7.1, another protein came on the scene: β‐arrestin is an adaptor protein downstream of GPCRs which traditionally is thought to play a role in GPCR internalization. But β‐arrestin is also thought to be able to mediate G‐protein independent signalling and is a key regulator of ion channels.^49^ Though there is not repeated evidence supporting that the AgRP binding can make MC4R recruit β‐arrestin to open Kir7.1, the demonstrated fact is that MC4R activation/deactivation does trigger Kir7.1 closure/opening, and the K^+^ efflux should be related to hunger signal release.^50^ Interestingly, a study in 2019 investigated a group of people who hold MC4R gain of function (GoF) variants that are associated with their significant lower BMI than normal people. They found the reason of this variant to protect them from obesity if that the GoF significantly increased the signalling bias towards β‐arrestin recruitment and PLC‐PKC pathway activation.^51^ So together with other found evidence, even though a lot more research is needed to clearly demonstrate their relations, a possible conclusion can be inferred that MC4R activation via specific biased agonists can inflict signalling on β‐arrestin recruitment which is related to Kir7.1 closure and appetite control, while AgRP binding to MC4R reverses this process (may/may not through β‐arrestin signalling), leading to Kir7.1 opening and hunger. The function of MC4R on PVH is shown in Figure 2.

For a long time, ligands have been developed and designed based on potencies tested on Gα_s_‐PKA biological assays for MC4R. Many of them were initially designed to selectively activate MC4R and inhibit appetite. Unfortunately, even though many of the MC4R agonists did show the anticipated appetite control, they at the same time showed cardiovascular, nausea and sex arousal side effects as mentioned in above session. This can be summarized as their multiple(equivalent) ability in activating several G protein‐mediated pathways upon binding to MC4R, including at least Gα_s_ (cardiovascular effects, sex arousal) and Gα_q_(appetite). Some of the early developed MC4R agonists are considered to be balanced agonists according to their drug effects shown in vivo. For example, MTII has obvious appetite inhibition^52^ while possesses severe increased cardiovascular effects, vomiting and sex arousal.^11^ Bremelanotide (PT141) is the deaminated version of MTII and behaves similarly as MTII; however, it was FDA approved for hypoactive sexual desire disorder.^12^ While some of the MC4R agonists tends to be Gα_s_ biased ligands, for example LY2112688 is a MC4R selective agonist and was originally designed for obesity, showing no efficacy in appetite control while significant cardiovascular responses and erectile activity.^14^ THIQ(MK0493) is also a selective and potent MC4R agonist but showed no efficacy/little effect in appetite control/weight loss, while sex arousal was observed.^53^ MC4‐NN‐0453 was developed by Novo Nordisk for obesity treatment, but it has sex arousal disturbance, erections reported with no weight loss efficacy detected.^15^ However, among all those potential candidates, one MTII structurally based derivative, setmelanotide (RM493), stands out for no obvious cardiovascular side effects^54^ while maintaining a promising appetite control during clinical trials,^54^, ^55^ making it an excellent treatment for obesity patients and it was approved by the FDA in 2020. The reason for RM493 appetite inhibition without cardiovascular side effect results from its selectively activating the Gα_q/11_ pathway, and meanwhile, it has 20‐fold more selectivity binding to MC4R compared to α‐MSH.^56^ As mentioned earlier in this section, previous research has clearly revealed the relation between Gα_q/11_‐PLC pathway and appetite control. So, whether setmelanotide's special performance in the clinical trial is dependent on its Gα_q/11_ activation potency remained to be investigated. With the widely used cell systems employed for measuring MC4R activation, it has been challenging to show the engagement of other G‐protein signalling pathway, including Gα_q/11_ and Gα_i/o_. Clément et al.^56^ provided evidence using HEK293 cell‐based systems that incorporated reporter gene system, such as cAMP‐binding response element(CREB) and nuclear factor of activated T cells(NFAT) reporters, that MC4R agonists can exhibit a differential preference for MC4R signalling through one or the other G‐protein coupled pathways. In their study, 3 representative MC4R agonists α‐MSH, LY2112688 and setmelanotide were tested and found that the EC50 value for the Gα_s_ signalling was as follows: α‐MSH 23 ± 7 nM; LY2112688 14 ± 4 nM; and setmelanotide 3.9 ± 1.7 nM, indicating a similar Gα_s_‐PKA activating potential. However, in the PLC assay (through the NFAT reporter gene), the EC50 were as follows: α‐MSH 480 ± 260 nM; LY2112688 330 ± 190 nM; and setmelanotide 5.9 ± 1.8 nM, showing an excellent Gα_q/11_‐PLC activation potency of setmelanotide than the other 2. Meanwhile in this assay, 100 nM of AgRP could even not antagonize setmelanotide stimulated PLC activation while the stimulation by α‐MSH and LY2112688 was successfully antagonized, indicating setmelanotide's strong signalling bias on PLC‐PKC cascade which is related to Gα_q/11_ recruitment.^56^

## 5 EVOLUTIONARY UNDERSTANDING OF THE WORKING MECHANISM OF GPCRs AND MC4R

**FIGURE 3:**
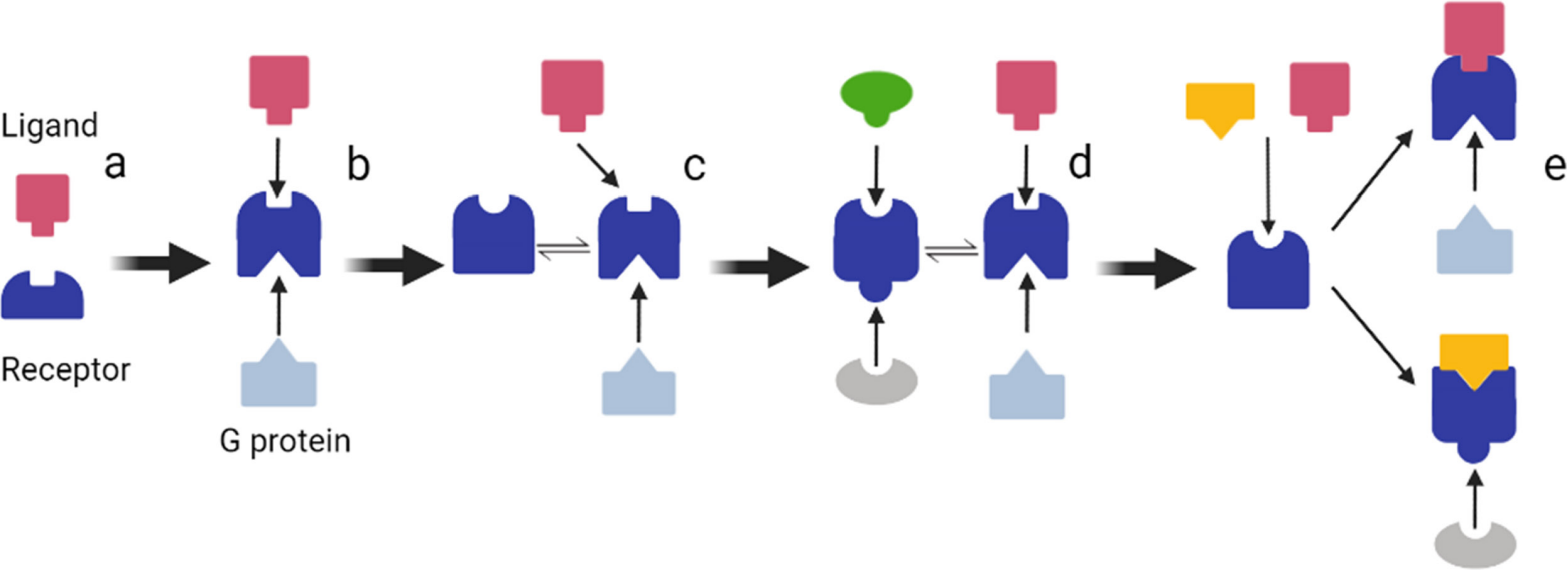
Level of complexity of complexity in different pharmacological models. (A) Classical Model, ligand simply binds to receptor to activate it. (B) Ternary complex model: The transducer(G‐protein) can bind to receptor after ligand binding and stabilize the whole receptor. (C) Extended ternary complex model: The receptor swings between an equilibrium of inactive(left) and active(right) form, the ligand and transducer have higher affinity binding with the active form. (D) Cubic ternary complex model: The cell membrane has multiple receptor conformations coexisted for binding with different ligands and transduces. (E) Multi‐state model: The receptor conformation can be induced by different ligands and thus be able to further bind with different transduces

The models for GPCR activation and signalling have been evolved over nearly a century. During early time, receptor activation was explained in Clark's classical model, in which the GPCR works as a lock and the ligand as a key to open this lock.^57^ With the understanding of GPCR's complexity going deeper, ternary complex model was proposed.^58^ Under this model, there are three principal components to initiate signalling: ligand, receptor and transducer(e.g. G proteins for GPCRs).^59^ These three components interact synergistically: Ligand binding can stabilize the receptor's conformation to bind with transducers, and the transducer binding to the receptor can in turn stabilize the ligand's binding to receptor.^59^, ^60^ Later, the ternary complex model was extended on the basis of previous theory, saying that receptor has 2 equilibrated states: active form and inactive form. In this conventional two state model, the inactive state is incapable of signalling, while the active state can recruit and functionalize transducers.^61^ Under this binary function concept, the receptor is modelled as a switch, with agonists stabilizing the ‘on’ state and antagonists stabilizing the ‘off’ state. However, some constitutively active mutants exhibit the capability for the receptor to trigger signalling without ligand binding,^62^, ^63^, ^64^ indicating that receptors are not necessarily locks waiting for keys. In this respect, cubic ternary complex (CTC) model was proposed. The CTC model represents a membrane system consisting of multiple receptor types(conformations) that interact with a diverse set of transducer molecules (G‐proteins) and ligand molecules (hormones). In this model, each receptor form is allowed to bind to only one G‐protein and/or hormone at a time, but different receptors are allowed to compete for G‐proteins and ligands. Thus, G‐proteins and ligands are envisioned as forming a common pool accessible to each receptor.^65^ The CTC model system has been pretty much like the current accepted GPCR working model in terms of its potentials in forming different conformations for multiple ligands binding and therefore triggers different signalling pathways. However, this model is still established without dynamic considerations that the different receptor conformations coexist when reaching equilibrium instead of dynamically changing upon binding to different ligands. Instead of encoding binary ‘on’ or ‘off’ signals, a more appropriate description for GPCRs is that it should act as an allosteric microprocessor with pluri‐dimensional efficacy and respond to different molecules with different transducer coupling efficiencies.^66^ Gradually, the multi‐state model of receptor activation was widely accepted due to the evidence obtained from many different pharmacological studies. Different from the previous ternary complex model in which receptor has a single signalling‐competent conformation resulting in activation of all signalling pathways, the multi‐state model highlights that receptor activation is a highly dynamic process in which multiple active conformations can be induced by different molecules to mediate different signalling pathways.^66^, ^67^ The evolution of the GPCR working mechanism is shown in Figure 3.

Biased signalling, as an important concept in GPCR intracellular signalling, has been identified for many members of the GPCR family.^68^ As a member of GPCRs, MC4R is also able to selectively stabilize particular receptor active conformations and preferentially triggering distinct signalling pathways, consistent with the multi‐state model of receptor activation and biased signalling in other GPCRs.^38^ The biased agonism, that different ligands can induce unique receptor conformations for distinct biological processes, is supported by numerous structure–function and pharmacological studies. Basic and translational studies conducted within the past years have led to an explosion of promising compounds with putative biased signalling and suggested that the therapeutic potential for biased GPCR ligands is profound.

## 6 THE STRUCTURE OF MC4R BOUND WITH SHU9119 AND SETMELANOTIDE REVEALS THE IMPORTANT TOGGLE SWITCH IN THE MC4R ORTHOSTERIC‐BINDING SITE

Despite the increasingly appreciated need to impart receptor bias on newly designed compounds, rational design of ligands remains difficult. Theoretically, rational design of a ligand to a specific receptor starts from knowing the conformation and property of the receptor so that the designed molecule can best fit and interact with the binding site. This will largely increase the success rate to make a potential drug candidate especially for the MC4R, as we discussed above, whose conformation is highly dynamic and closely related to the selection of downstream signalling. In the usual model, ligands binding at the orthostatic region of a GPCR control the differential interaction at its intracellular region with different effectors. Within this paradigm, the structural understanding has generally been limited to efforts at rationalizing the binding differences between differently biased ligands as well as investigations of protein structural and dynamics processes which may be involved in receptor trafficking. For melanocortin receptors, the challenge to find biased ligands is further exacerbated by the absence of corresponding GPCR structures.^69^ In this respect, the MC4R structures bound with the several canonical biased/balanced agonists are greatly needed, as these structures can to some degree give inspirations about how to design the appropriate ligands to induce an expected receptor conformation so that the desired signalling occurs. These studies (docking, molecular dynamic simulations and so on) are now commonly used in computational chemistry or biochemistry of virtual drug design and screening. However, as a membrane‐integrated GPCR, MC4R is highly mobile, and its conformation largely depends on the support of the phospholipids around it, making it hard to be crystallized. The MC4R structure was not determined until 2020. Yu et al. determined the structure of MC4R co‐crystalized with the antagonist SHU9119, giving the conformation of the MC4R at its ‘rest state’.^70^ In the multi‐state model, different ligands induce and strengthen different MC4R conformations upon binding, making it more capable in recruiting a specific G protein/β‐arrestin/Kir7.1. Thus, theoretically speaking, binding with an antagonist will induce a MC4R conformation very much like its resting state because the antagonist binding triggers no observable biological change to the downstream signalling, indicating no crucial conformational change. To stabilize the structure, they mutated several key sites of the wild‐type MC4R, together with truncation of the N terminal and C terminal residues as well as Pyrococcus abyssi glycogen synthase (PGΑS) inserted into the receptor's third intracellular loop (ICL3).^70^ These modifications stabilized MC4R without disturbing the activity. In this study, they found the importance of the Calcium moiety in helping with ligand binding at the MC4R orthostatic‐binding site, and that the coupling of Kir7.1 is highly dependent on the ligand binding. Because when they mutated D122 to alanine, the coupling of Kir7.1 to MC4R disappeared whereas D122 has long been demonstrated to be crucial in agonist binding and biological activity.^71^, ^72^ This finding supported previous studies that the PVH depolarization/hyperpolarization mediated by Kir7.1 is highly related to the activity of the MC4R.

**FIGURE 4:**
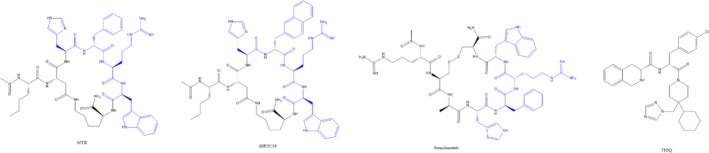
Chemical structures of 4 representative MC4R‐binding ligands. MTII is a non‐selective MC4R balanced agonist with cardiovascular side effects and sex arousal upon binding to MC4R for appetite control. SHU9119 is a MC3R and MC4R antagonist but MC1R and MC5R agonist, its only difference from MTII is the change of D‐Phe to D‐Nal(2′). Setmelanotide is a potentially biased MC4R agonist with no cardiovascular side effects while good appetite inhibition upon binding to MC4R. THIQ is a small molecule MC4R selective agonist with obvious sex drive and cardiovascular effects but little on appetite. The H‐F‐R‐W pharmacophore is labelled in blue

**FIGURE 5:**
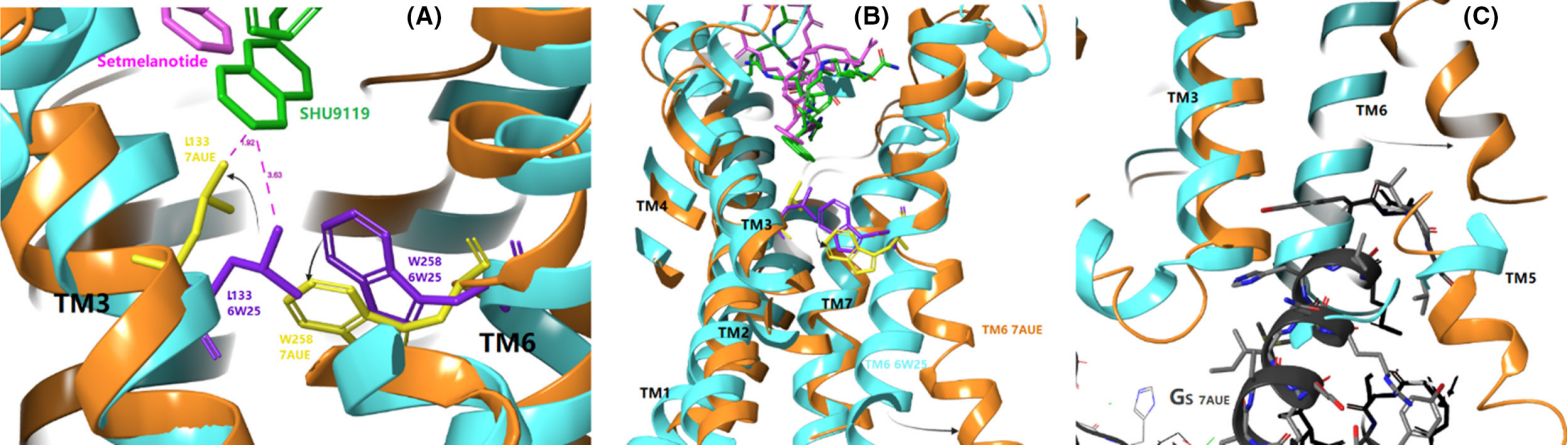
Comparison of MC4R crystal structure 6 W25 and 7AUE. (A)Superposition of the MC4R active complex (7AUE orange) with an antagonist bound receptor (6 W25, light blue). Setmelanotide(Pink) and SHU9119 (Green) bound at the MC4R canonical pocket differentially interact with L133 and W258 (yellow on 7AUE and purple on 6 W25) composes the switch that controls receptor conformation. (B)The bulky D‐Nal(2′) side chain does not have clash with L133 at resting state, maintaining W258 upwards and TM6 straight. When changing to setmelanotide, the naphthyl group is changed to a phenyl group, leaving space for L133 to stick up, which pushes W258 to bend down and thus force the TM6 to stick outwards. (C)The outward movement of TM6 makes room for Gα_s_ (showing in black ribbon and dark grey tubes for residues). *Resource is from PDB‐6 W25 and PDB‐7AUE, more specific illustration can be retrieved from Israeli. et al. Science. 2021^73^

This year 2021, Israeli et al.^73^ figured out the structure of MC4R binding with setmelanotide(RM493) and G protein complex through Cryo EM. In this research, the full structure of an activated complex was clearly shown, and most importantly, the mechanism of MC4R activation was firstly ‘seen’ via the alignment and comparison of this activated conformation and the previous SHU9119 bound non‐activated conformation. This comparison is shown in Figure 5. The significance of this study is that it for the first time revealed the identity of the key sites related to agonism in the MC4R‐binding pocket and provided solid evidence showing the many previous findings about how the modifications at the D‐Phe4 position in all His‐D‐Phe‐Arg‐Trp pharmacophore based α‐MSH derivatives affects the MC4R activation.^74^, ^75^ It is been an interesting finding for many years that SHU9119 surprisingly switched the MC4R agonist to a MC4R antagonist, which contains D‐Nal(2′) switched from D‐Phe compared to MTII (same as Setmelanotide, they are both cyclized α‐MSH derivative with the same His‐[D]Phe‐Arg‐Trp pharmacophore and similar ring size), structure of these 3 compounds is shown in Figure 4. This brought a lot of inspirations to ligand development for MC4R. Modifications to adjust the size of the D‐Phe side chain were diversly applied, and one most impressive change was the para halogenation with F, Cl, Br and I.^75^ It turned out that the MC4R activation potency was increased a bit by the addition of F and much more with Cl, then decreased with the Br and finally totally abolished with I. It is clear that as the atom size increases, Cl gives an optimal size for the receptor activation whereas Br and especially I seem to be too big so that the para Iodine gave similar effect as DNal(2′). Even though in the past decades, MC4R drug development was successful due to the accumulated experience from lots of ligands and structural activity relationship (SAR) studies, the overall drug design was empirical and this switch of agonism to antagonism phenomenon due to D‐Phe site modification has been seeking a deep explanation until this year. The Israeli teamwork gave convincing evidence from their cryo‐EM generated MC4R structures, showing that this conversion is due to the D‐Phe side chain is inserted into the deep pocket and modulates L133 on TM3 and W258 on TM6(Figure 5). Simply speaking, the smaller side chain of D‐Phe makes more space for L133 to stick up, thus in turn pushes W258 down and out, making the bottom half TM6 helix sticking out and, in this way exposes the Gα protein‐binding site as shown in Figure 5. This work explained the previous observed phenomenon that substituting the residue L133 to methionine led to the complete conversion of SHU9119 activity from antagonist to agonist of MC4R,^71^ which is possibly owing to the fact that the methionine side chain has more freedom to adopt different rotamers accommodating the bulky D‐Nal residue of SHU9119. In summary, both studies provided solid reasoning why His‐Phe‐Arg‐Trp pharmacophore is necessary in all MSH derivatives to fulfil function. In addition, the MC4R structures give instructional information of key amino acids which interactions need to be taken into consideration when designing ligands for the MC4R. For example, the L133, L258 and those canonical ones which already had been demonstrated many years ago over many studies, for example D122, D126 and E100.^77^, ^78^ Similarly, both studies observed an almost identical location of the single calcium moiety around these 3 amino acids, forming a salt bridge to connect these MC4R activity essential amino acids on TM2 and TM3 to the bound ligands.

## 7 THE MC4R STRUCTURES BOUND WITH SEVERAL REPRESENTATIVE LIGANDS REVEAL THE POSSIBLE LIGAND–RECEPTOR INTERACTIONS RELATED TO SIGNALLING BIAS

**FIGURE 6:**
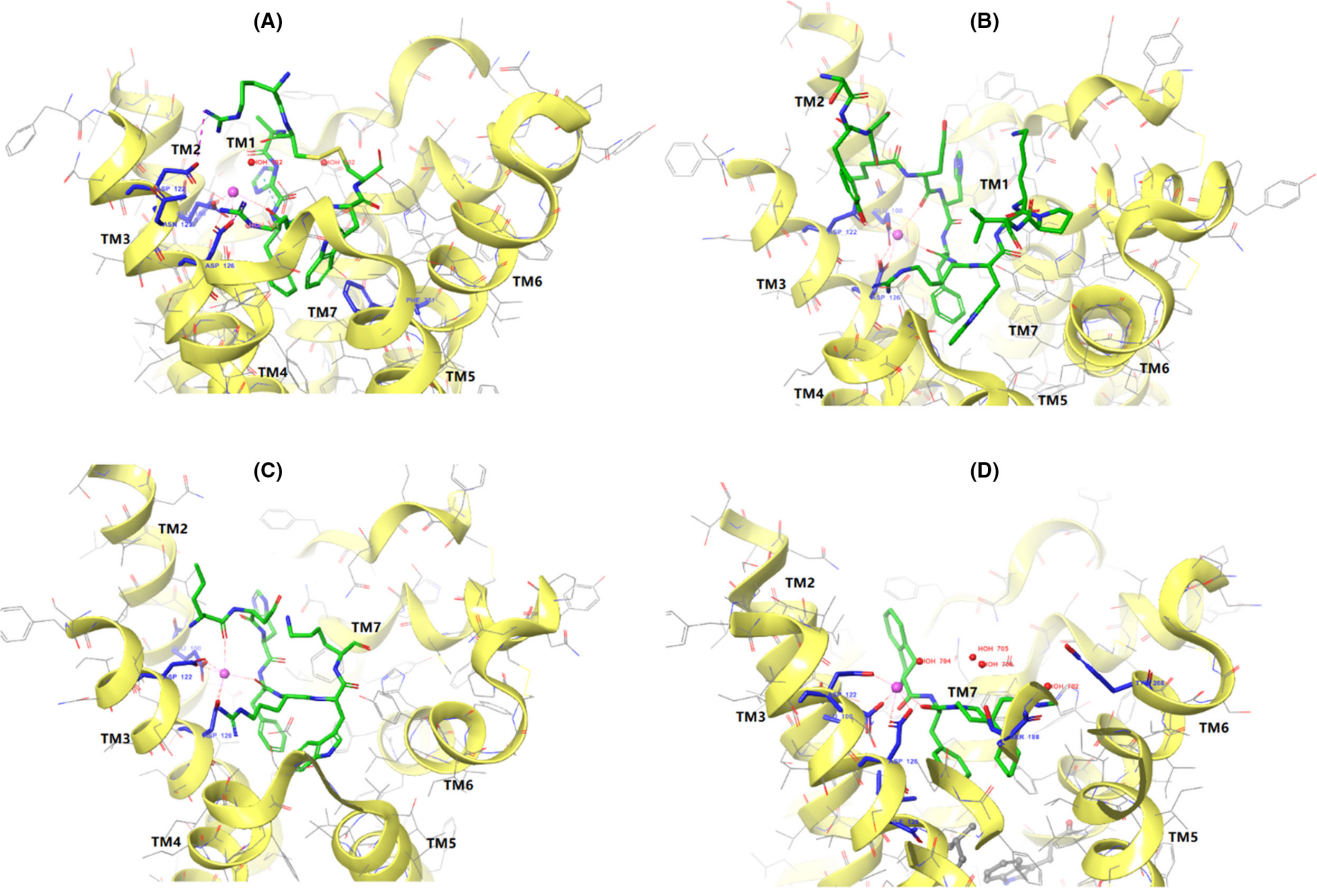
Binding modes of 4 representative agonists to MC4R. MC4R is shown in yellow ribbon style, ligands are shown in green stick style and calcium is shown in pinkish purple ball style. Key amino acids on MC4R interacting with Ca^2+^ and ligands are shown in blue stick style. Water molecules are shown in red ball style. A: Setmelanotide binding with MC4R, PDB: 7PIU. B: NDP‐α‐MSH binding with MC4R, PDB: 7F54. C: Bremelanotide (deaminated MTII) binding with MC4R, PDB: 7F55. D: THIQ binding with MC4R, PDB: 7F58. More specific illustrations can be retrieved from Heyder. et al. Nature. 2021^79^ and Zhang et al. Nature. 2021^82^

As discussed above, setmelanotide has been demonstrated as a biased MC4R agonist that mainly turns on the Gα_q_‐PLC pathway. Even though Israeli et al.^73^ revealed the key mechanism that controls the opening of the allosteric site for Gα_s_ protein binding, it is still not clear why setmelnaotide can induce MC4R's preference more on Gα_q_ signalling. Later this year 2021, Heyder et al.^79^ resolved the MC4R structures binding with setmelanotide (Gα_q_ signalling biased agonist) and NDP‐α‐MSH (balanced agonist), together with the G protein bound. Most of the interactions of the MC4R‐binding pocket with setmelanotide and NDP‐α‐MSH are very similar, because they are all _Gαs_ bound MC4R complex and should theoretically be similar in conformation, as explained in earlier sections. There are several differences between the 2 structures: 1. The setmenlanotide D‐Phe has a hydrophobic interaction with F261 on TM6, pushing it more outward compared to the NDP‐α‐MSH complex. 2. In the setmlanotide complex, D122 is fully oriented towards Ca^2+^. Secondly, the side chains of Arg(R6) of both ligands showed slightly different orientations in the 2 structures. Setmelanotide has a special interaction with N123 on MC4R and this structural difference is accompanied by a slight horizontal TM3 shift in the setmelanotide–MC4R complex, leading to a ligand‐dependent Ca^2+^ positioning. 3. In contrast to the NDP‐α‐MSH complex, D122 is fully oriented towards Ca^2+^. 4. The setmelanotide interaction between the first arginine(R1) and D122 reduces the number of interactions with the cofactor Ca^2+^ involved in stabilizing the peptide‐TM2‐TM3 interface with a fourfold coordination of the ion in contrast to the fivefold coordinated Ca^2+^ in the NDP‐α‐MSH‐bound receptor. The reduced number of setmelanotide interactions is also related to a double conformation of E100, in which one conformation participates in a hydrogen bond network with a water molecule and H40 on MC4R. These differences together make the 2 essential amino acids T150 and H158 in the allosteric G protein‐binding site different in these 2 complexes, providing a possible reason for the setmelanotide's bound MC4R recruiting the Gα_q_ protein. The binding patterns of both ligands to MC4R are shown in Figure 6.

Another representative MC4R selective ligand is THIQ (Figure 4) which is a small molecule developed by Merck. It has highly selective potency on MC4R, which is 1400‐fold vs MC1R, 1200‐fold vs MC3R and 360‐fold vs MC5R.^80^ However, previous animal studies reported that THIQ stimulates little effect on appetite or inflammation but can strongly activate sexual activity.^81^ This is an indication of its capability of biased signalling of the Gα_s_ pathway. Similarly, this year 2021, Zhang et al.^82^ resolved the MC4R structure bound with G protein and 4 representative agonists including α‐MSH, NDP‐α‐MSH, Melanotan II(MTII) and THIQ, which binding patterns are shown in Figure 6. The other 3 share similar binding patterns and interactions except THIQ, which is specifically recognized by the MC4R orthosteric‐binding site. Compared to the peptidic agonists listed above, the conformational architecture of THIQ reassembled the His‐Phe‐Arg‐Trp pharmacophore of α‐MSH. However, in contrast to peptide agonists, only one carbonyl oxygen atom from THIQ, which is equivalent to that of the Phe7 in endogenous α‐MSH, participated in coordinating Ca^2+^. Hence, it appears that the function of THIQ is less dependent on the calcium compared to all the other peptide ligands, which led to a fairly large difference in the binding mode to MC4R. In addition, THIQ has special interactions with those that go beyond the conserved amino acids as binding determinants including TM3 residue I129, extracellular loop (ECL)2 residue S188 and TM6 residue Y268.^74^ These differences may account for the biased Gα_s_ signalling controlled by THIQ.

## 8 FUTURE DIRECTIONS

In the past decades, MC4R research has been blooming, making this receptor the most well‐known of the melanocortin receptors. So far, the understanding of MC4R working mechanisms is revealed step by step. The discovery of alternative GPCR signalling pathways provides inspirations for novel drug screening beyond technologies that focus solely on proximal signalling responses mediated by the Gα_s_‐PKA pathway. According to the research so far, the toggle switch deep inside the MC4R orthosteric‐binding site is of great significance to be taken into consideration during drug design, because the ligand interaction at this site directly determines if the allosteric G protein‐binding site can be opened. The past decades have witnessed waves of peptide drug development from the mimics of natural endogenous hormones to modification of the humanmade mimics, and from the non‐selective ligands to receptor subtype selectivity. As the knowledge of cell biology progresses, people have now been clearer about the reasons for many of the side effects and the way to get rid of them should be the next wave of drug design targeting specific cell signalling. For the MC4R, targeting the non‐canonical signalling pathways depending on the physiological response desired could theoretically improve current MC4R‐based therapies through enhanced efficacy and reduced side effect profiles. It is fairly clear that the different ligands binding to MC4R induce specific receptor conformations that favour specific G protein subtypes. This conclusion is in alignment with our own research on Plasmon Waveguide Resonance (PWR) spectroscopy, where the conformational difference can be detected and converted into the position of the absorbed laser peak. Our result also showed similar phenomenon that the balanced agonists, for example NDP‐α‐MSH and MTII, induce similar MC4R conformations and THIQ and setmelanotide, as biased ligands, trigger different MC4R conformations while the antagonist SHU9119 leads to almost no change of the MC4R structure, explaining its role as an antagonist. However, the number of structures observed for MC4R is still limited, with only several representative ligands co‐crystallized (MSH, NDP‐α‐MSH, MTII, THIQ and setmelanotide). Thus, it is still not clear if the positional differences of those specific amino acids, between these MC4R structures, at the orthosteric‐binding pocket and at the allosteric G protein‐binding site have definite correlation. In other words, it is still not completely clear about the universal rule of the G protein profiling that MC4R and its ligands control. In this respect, more representative biased ligands, for example the typical Gα_q_ biased ligands or Gα_s_ biased ligands for MC4R need to be developed, so that more co‐crystal structures with these ligands can give conformational information for the specific biased signalling. The rule of the signalling pathway selection can to some degree be summarized and utilized in the future drug design. This is also of great significance to reveal the secret of the biased pathway selection happening on other MCR subtypes because they share a lot of conformational similarities and similar activating mechanisms may also apply. Further, as a GPCR, the findings on MC4R‐biased signalling selection may also be instructive for the studies of other GPCRs with important biological functions for example the Opioid receptor family.

In addition to agonists and antagonists, inverse agonists, like AgRP and its mimics for MC4R, are also an important area for biased signalling research, which represents a totally novel signalling type than the currently discussed pathways. Making clear the binding pattern of AgRP with MC4R may help reveal the key mechanism of Gα_i_ biased signalling. Beta‐arrestins can also regulate special signalling on MC4R which is independent from the G protein‐mediated regulation. Another interesting phenomenon is that setmelanotide does incur Gα_s_ recruiting as observed in Gα_s_‐PKA based cell assays and the resolved setmelanotide‐MC4R‐Gα_s_ crystal structure. However, it has very strong Gα_q_ activation potency which other MC4R agonists do not have. This brings a new question of interest: Does setmelanotide activate Gα_q_ more than Gα_s_ or not? According to clinical trials, it apparently showed that Gα_q_ signalling is dominating because there of no observable cardiovascular side effects. This may require further research and the quantitative evaluation of the signalling strength between these 2 pathways being established. In conclusion, even though there have been recent novel findings on MC4R structures, except the one co‐crystallized with SHU9119, all the others are Gα_s_ bound complexes which is still different from binding with Gα_q_. Thus, the setmelanotide‐induced Gα_q_ biased activation mechanism is still not totally clear and more like a hypothesis based on current evidence. Given the significant economic and time costs in late phase clinical trials, accurately quantifying the biased signalling properties in the early phase of drug development is necessary. This requires more biological assays targeting different signalling pathways with accuracy and dependability. The overall progress in MC4R will at the same time push forward the resolution of structures of other melanocortin receptors due to their high similarity of sequence and conformation, which will further benefit the drug development on MCR family and diseases related to these receptors.

## AUTHOR CONTRIBUTIONS

**Zekun Liu:** Writing – original draft (lead); writing – review and editing (lead). **Victor J. Hruby:** Supervision (lead).

## CONFLICT OF INTEREST

The authors declare no conflicts of interest.

## Data Availability

The data that support the findings of this study are available in [repository name] at [URL/DOI], reference number [reference number]. These data were derived from the following resources available in the public domain: [list resources and URLs]
